# OP02 Osseointegrated Prosthetic Implants (Direct Skeletal Attachment) For People With Lower-Limb Amputation: A Hospital-Based Health Technology Assessment

**DOI:** 10.1017/S0266462324000667

**Published:** 2025-01-07

**Authors:** Elena Galfrascoli, Dario Concetto Pistritto, Maristella Ghiringhelli, Martina Sterpetti, Michele Bertoni, Silvia Bozzi, Federica Asperti, Raffaella Cavi

## Abstract

**Introduction:**

Patients with amputation need a safe and comfortable connection with the prothesis. Traditional sockets may lead to skin tearing, pain, and limitation of movement. Osseointegrated protheses connected to residual bone may have a positive impact on patients’ quality of life. Our research question: Are there economic and organizational benefits from the introduction of a direct skeletal attachment (DSA) procedure, as well as clinical benefits?

**Methods:**

Firstly, a systematic literature review was carried out on PubMed and Scopus, and the Critical Appraisal Skills Programme (CASP) tool was applied to assess the evidence’s quality. A health technology assessment (HTA) evaluation was conducted (EUnetHTA, 2016), adopting the perspective of a public hospital in Italy, comparing two scenarios—traditional and innovative—related to traditional protheses and the DSA approach. Seven experts (orthopedic surgery, physical medicine, and rehabilitation and physiotherapy) were involved for the administration of a qualitative questionnaire. For the economic evaluation, a cost-effectiveness analysis and budget impact analysis were defined. Finally, a multiple-criteria decision analysis was performed.

**Results:**

The literature search yielded 314 citations published until December 2022: eight were eligible for analysis. Three were the system analysed: ‘OPRA’, ‘ISP’ and ‘OPL’. The efficacy of the systems is linked to a better distribution of bone stress: an increase in bone mineral density was recorded near the implants (respectively 28%, 27%, and 18% – after 60 months). The safety of DSA depends on the design and integrity of the connection between tissues and implant. The impact on the budget is an increase of 27 percent in costs for each patient treated. Concerning the social and ethical implications, DSA results in the preferable approach (1.48 vs 0.34), as it can limit social costs (0.29 vs −0.29).

**Conclusions:**

The comparative evaluation was carried out using a scoring method: the main advantages related to innovative prostheses are based on effectiveness and safety, as well as social impact and organizational impact, especially due to the ability of the prostheses to reduce the risk of adverse events and long rehabilitation, with important clinical benefits and organizational savings for the hospital management.

